# Trogocytosis represents a novel mechanism of action of daratumumab in multiple myeloma

**DOI:** 10.18632/oncotarget.26098

**Published:** 2018-09-14

**Authors:** Jakub Krejcik, Niels W.C.J. van de Donk

**Affiliations:** Amsterdam UMC, Vrije Universiteit Amsterdam, Department of Hematology, Amsterdam, The Netherlands

**Keywords:** multiple myeloma, immunotherapy, daratumumab, CD38, trogocytosis

Daratumumab is a fully human CD38-targeting monoclonal antibody (mAb) with pleiotropic mechanisms of action, and is approved as monotherapy and in combination with standards of care for the treatment of multiple myeloma (MM). In preclinical models, daratumumab elicited cell death mainly through antibody-dependent cell-mediated cytotoxicity (ADCC), antibody-dependent cellular phagocytois (ADCP), and complement-dependent cytotoxicity (CDC) [1]. These Fc-dependent immune effector mechanisms are classic mechanisms of action of monoclonal antibodies used for the treatment of cancer.

We showed that high CD38 expression levels on the MM cell surface are important for effective CDC and ADCC, which explains that clinical response to daratumumab monotherapy is partly dependent on baseline CD38 expression levels on the tumor cells [2]. Nevertheless, we have recently shown that daratumumab treatment leads to a reduction of CD38 levels on MM cells within a few hours after the first infusion. The mechanisms of daratumumab-mediated CD38 reduction on MM cells include both the selection of cells with lower CD38 expression, as well as uptake of CD38-daratumumab complexes by monocytes and granulocytes in the process of trogocytosis. Trogocytic transfer of CD38 from the MM cell surface to effector cells may reduce the ability of daratumumab to kill MM cells via CDC and ADCC, thereby compromising the therapeutic efficacy of daratumumab. However, this phenomenon of CD38 reduction occurs early in the treatment, is a uniform response in all patients treated with daratumumab, and includes also patients with sustained clinical responses and those with an increasing depth of response over time. Therefore, CD38 reduction via trogocytosis should not necessarily be considered as an escape mechanism from daratumumab treatment, but in fact trogocytosis may be beneficial and represent a novel mechanism of action of daratumumab, particularly when the classic Fc-dependent immune effector mechanisms (ADCC, CDC, and ADCP) are limited in their capacity to kill MM cells as a result of daratumumab-mediated CD38 reduction on the MM cells [3].

Interestingly, in the process of trogocytosis there is not only transfer of CD38-daratumumab complexes, but also tumor cell membrane fragments are transferred to effector cells. This provides an explanation for the daratumumab-mediated reduction of several other important adhesion proteins on the MM cell surface such as CD56, CD49d, and CD138 [3]. The decreased expression of these adhesion molecules may impair the ability of the MM cell to interact with the protective bone marrow microenvironment, thereby reducing microenvironment-derived survival signaling as demonstrated in a mouse model of CD38-expressing chronic lymphocytic leukemia [4]. This mechanism of action may partly explain the strong clinical synergy observed between daratumumab and other routinely used anti-MM agents, whose activity is reduced by the protective micromilieu [5].

Furthermore, CD38 is an immunomodulatory molecule, which inhibits T-cell function via adenosine receptor signaling [6]. It is therefore conceivable that down-regulation of CD38 on both MM cells and cells of the tumor microenvironment by trogocytosis, may lead to an improved adaptive immune response against MM cells. In addition, daratumumab eliminates CD38-positive immune suppressor cells including regulatory T-cells, regulatory B-cells, and myeloid-derived suppressor cells [7]. Altogether, daratumumab treatment results in a less immunosuppressive microenvironment, which explains that patients treated with this antibody experience an expansion of T-cells in both peripheral blood and bone marrow [7]. Furthermore, during daratumumab treatment, specific CD8+ subpopulations are altered, including a significant decrease in naive T-cells and increase in effector memory CD8+ T cells, indicating a shift in effector T-cells towards an antigen-experienced phenotype with immunological memory and possible reactivity against tumor antigens [7]. Importantly, daratumumab treatment also significantly increases T-cell clonality [7]. The increase in T-cell clonality is greater in patients with a good clinical response, and is correlated with the increase in CD8+ T-cells. In addition to increased T-cell clonality, patients with a response to daratumumab demonstrate increased T-cell responses to preexisting viral- and alloantigens, suggesting that daratumumab treatment is able to reverse immune suppression in MM and leads to antigen-driven T-cell expansion which may be responsible for tumor control [7].

Many other investigational monoclonal antibodies targeting diverse antigens expressed on MM cells showed impressive ability to induce CDC and ADCC against MM cells in vitro. However, when clinically tested, they were not effective as single agents and even their combination with standard therapy did not always provide substantial clinical benefit. On the contrary, all currently available CD38-targeting antibodies (daratumumab, isatuximab, and MOR202) have demonstrated marked anti-MM activity alone and in combination with other agents [8]. Reversal of immune suppression by decreasing the amount of CD38 molecules on the tumor cells and in the tumor microenvironment by trogocytosis may be a crucial mechanism of action of daratumumab in MM. The unique immunomodulatory characteristics of the CD38 target molecule and its downregulation by daratumumab-mediated trogocytosis may actually be one of the explanations that daratumumab is the first monoclonal antibody with single agent activity in MM [8].
